# Time-resolved X-ray and XUV based spectroscopic methods for nonadiabatic processes in photochemistry

**DOI:** 10.1039/d2cc04875b

**Published:** 2022-10-21

**Authors:** Thomas Schnappinger, Deependra Jadoun, Mahesh Gudem, Markus Kowalewski

**Affiliations:** Department of Physics, Stockholm University, Albanova University Centre SE-106 91 Stockholm Sweden markus.kowalewski@fysik.su.se

## Abstract

The photochemistry of numerous molecular systems is influenced by conical intersections (CIs). These omnipresent nonadiabatic phenomena provide ultra-fast radiationless relaxation channels by creating degeneracies between electronic states and decide over the final photoproducts. In their presence, the Born–Oppenheimer approximation breaks down, and the timescales of the electron and nuclear dynamics become comparable. Due to the ultra-fast dynamics and the complex interplay between nuclear and electronic degrees of freedom, the direct experimental observation of nonadiabatic processes close to CIs remains challenging. In this article, we give a theoretical perspective on novel spectroscopic techniques capable of observing clear signatures of CIs. We discuss methods that are based on ultra-short laser pulses in the extreme ultraviolet and X-ray regime, as their spectral and temporal resolution allow for resolving the ultra-fast dynamics near CIs.

## Abstract 2

Novel spectroscopic techniques based on ultra-short laser pulses in the X-ray regime are reviewed, capable of observing clear signatures of conical intersections.

## Graphical abstract

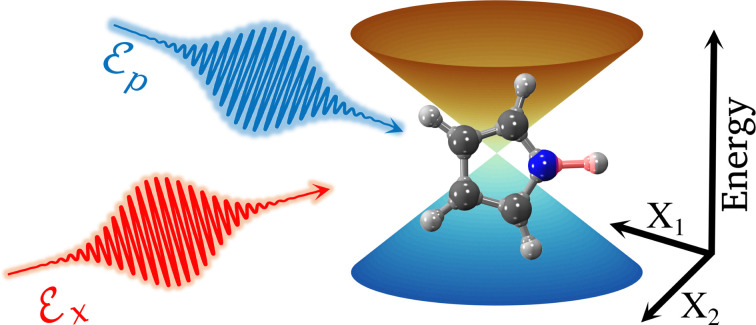

## I. Introduction

The interaction of visible or ultraviolet (UV) photons with a molecular system is a fundamental step of various chemical, physical, and biological phenomena such as the primary event of vision,^1,2^ photosynthesis,^3–5^ vitamin D synthesis^6–8^ and different types of photocatalysis.^9–12^ Photoexcited molecules can undergo photochemical reactions and decay back to the ground state without the emission of photons, by means of nonadiabatic decay channels. If the spin state is not changed by this transition, the process is called internal conversion (IC) and is happening mainly in the vicinity of an avoided crossing or a conical intersection (CI). The latter are extraordinary points, seams or even higher-dimensional spaces in a molecular system where the involved electronic states become degenerate, allowing an ultra-fast and very efficient population transfer between these states mediated by nonadiabatic coupling (NAC) elements. However, the influence of a CI on the dynamics of a molecule goes far beyond being a simple funnel. The Born–Oppenheimer approximation (BOA) breaks down in the vicinity of a CI, and a strongly mixed dynamics of electrons and the nuclei occurs.^13–16^ For example, a molecular wave packet approaching a CI gains a non-trivial geometric phase, also called the Pancharatnam–Berry phase,^17,18^ which can promote self-interference.^19–22^ In general, a CI creates a coherent superposition of electronic states whose composition is determined by the shape and size of the NAC elements. The coherence lifetime of the superposition depends strongly on the nuclear dynamics^23–26^ which is directly influenced by the topography of the CI.

In recent decades, great theoretical progress has been made towards simulating strongly coupled electronic and nuclear systems at different levels of theory.^27–32^ By applying these methods, it has become possible to explain and partially predict the results of photochemical reactions.^33–40^ Simultaneously, the observation^41–50^ of ultra-fast population transfer and the rapid change in properties, such as (transition) dipole moments, during photochemical processes has given experimental evidence of CIs being involved in the dynamics. With these joint experimental and theoretical efforts, the existence of CIs is now widely accepted. To gain even deeper insights into the ultra-fast dynamics close to a CI, novel spectroscopic and diffraction methods are proposed which might be capable of monitoring different features of nonadiabatic processes near a CI. In this context, an important aspect is the study of the induced electronic coherence, since it carries the information of the relative energy separation between the involved electronic states. The observation of quantum coherent oscillations in the signal, originating from the electronic coherence, could be a clear indication of the occurrence of a CI. However, it has to be taken into account that the electronic coherence itself is not an observable. It is defined as an overlap between nuclear wave functions in different electronic states and is thus a basis-dependent quantity. The emergence of features related to the generated electronic coherence in a spectrum depends on the chosen observable such as polarizability, transition dipole moment, or charge density.

The complex interplay of electronic and nuclear degrees of freedom, near a CI, makes it challenging for a single spectroscopic technique to disentangle the dynamics and different techniques maybe applied. Ultra-short pulses are necessary since the processes in the vicinity of a CI take place on an ultra-fast timescale and cover a broad energy range. X-ray pulses produced at modern free-electron laser (FEL) facilities or generated by high-harmonic generation (HHG) sources are capable of providing the required temporal and spectral resolutions and therefore are being explored as potential probes for nonadiabatic dynamics. Off-resonant X-ray Raman spectroscopic methods, such as transient redistribution of ultrafast electronic coherence in attosecond Raman signals (TRUECARS), have been theoretically demonstrated to be capable of studying the electronic coherence generated near a CI.^51^ Transient X-ray absorption spectroscopy (TXAS) has been used experimentally to detect the presence of CIs in molecules.^48–50,52^ Time-resolved X-ray spontaneous emission spectroscopy (XSES) has been theoretically demonstrated to visualise the intersecting electronic states near a CI.^53^ Time-resolved X-ray diffraction (XRD) has been implemented theoretically to spatially detect the occurrence of nonadiabatic dynamics in a molecule.^54–56^ Besides X-ray based methods, time-resolved photoelectron spectroscopy (TRPES) has been shown capable of observing the population transfer and studying the electronic coherence created in the vicinity of a CI.^57–60^

In this feature article, we want to give an overview of these novel time-resolved spectroscopic techniques that can be used to study dynamics in the vicinity of CI. After giving a brief introduction to CIs and short recap of the basic concepts of nonadiabatic dynamics induced by light–matter interaction, we will discuss the theoretical description of different time-resolved spectroscopic and diffraction signals. We will focus on techniques which allow the study of the electronic coherence generated near a CI.

## II. Theory

### A. Nonadiabatic dynamics

As a first step on the way to simulate spectroscopic fingerprints of a CI involved in the dynamics, one has to determine the molecular dynamics of the system by solving the time-dependent Schrödinger equation,1
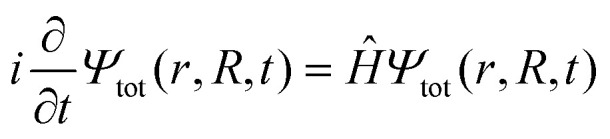
with *r* and *R* being the electronic and nuclear coordinates, respectively. Atomic units are used throughout the paper unless otherwise stated. The total Hamiltonian *Ĥ* consists of the kinetic energy operator *T̂*_N_ and the electronic Hamiltonian *Ĥ*_el_. The total molecular wave function *Ψ*_tot_(*r*,*R*,*t*) in the Born representation is formulated as a sum of product states2
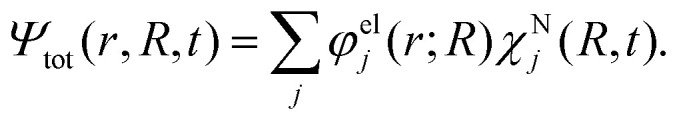
Here *χ*^N^_*j*_(*R*,*t*) are the time-dependent nuclear wave functions. The adiabatic electronic wave functions *φ*^el^_*j*_(*r*; *R*) are only parametrically dependent on *R* and are defined as the eigenfunctions of the time-independent electronic Schrödinger equation:

3 *Ĥ*_el_*φ*^el^_*j*_(*r*;*R*) = *E*_*j*_(*R*)*φ*^el^_*j*_(*r*;*R*)

**Fig. 1:**
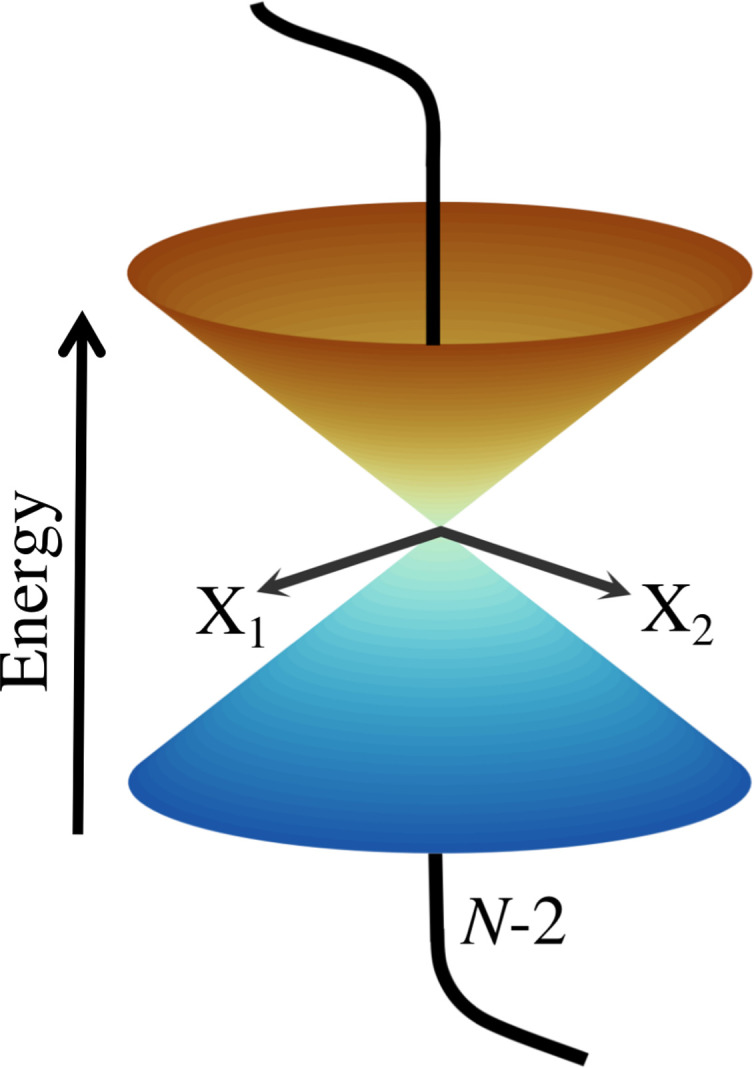
Schematic representation of two adiabatic PESs forming a CI in the two-dimensional branching space spawned by X_1_ and X_2_. Any motion within the branching space lifts the degeneracy and generates the characteristic cone shape, while the states stay degenerated in the *N* − 2-dimensional intersection space.

By solving the electronic Schrödinger equation for different nuclear configurations, the resulting eigenvalues, *E*_*j*_(*R*), define the potential energy surface (PES) of a given electronic state *j*. Inserting the adiabatic electronic wave functions in eqn (1) and subsequently integrating over *r* gives rise to the equation of motion for the nuclear part *χ*^N^_*j*_(*R*,*t*).4
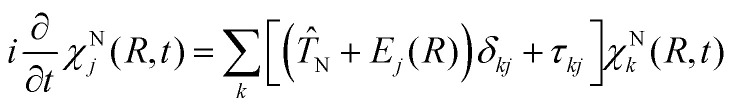
Here *T̂*_N_ takes the following form5
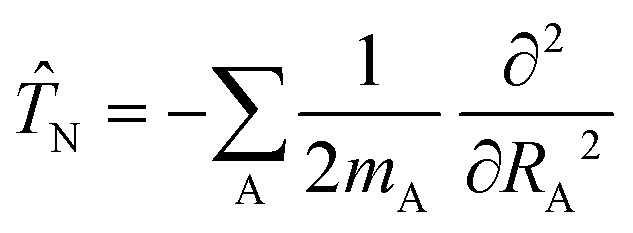
with *m*_A_ being the mass of atom A. The nonadiabatic coupling elements *τ*_*kj*_ can be expressed as:6
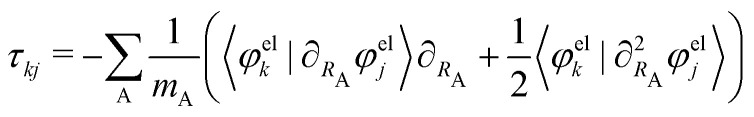
The first part of the summation contains the first derivative of an electronic wave function with respect to the nuclear coordinates, and thus the whole element *τ*_*kj*_ becomes a vector. These elements are neglected in the BOA^61^ and are partialy included in the Born–Huang approximation.^62^ It can be shown^16^ that *τ*_*kj*_ are inversely proportional to the energy gap between the two states *k* and *j*. Therefore, if the PESs are well separated, the BOA is valid and the nuclear dynamics can be described by a single electronic state. For situations where the energy gap between the electronic states is getting smaller, *τ*_*kj*_ can not be neglected and the BOA breaks down. In the extreme case of a vanishing energy gap, the two (or in principle more) adiabatic PESs form a CI (see Fig. 1) and the nonadiabatic coupling elements diverge to ±∞. It should be noted that non-vanishing *τ*_*kj*_ and CIs can occur only between adiabatic states of the same spin. For comparable degeneracies between electronic states of different spin, the situation is fundamentally different and can be associated with relativistic effects (spin–orbit-couplings).^63,64^

To give a proper definition of a CI and also to better describe its properties, we use a simple two-state model and reformulate eqn (4) as 2 × 2 matrix equation7
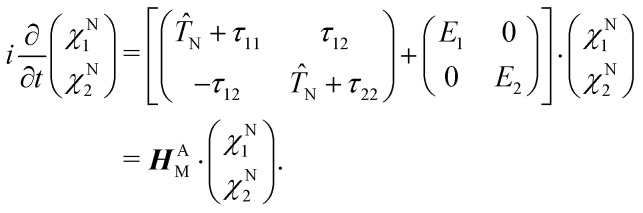
***H***^A^_M_ is the matrix that describes the bare molecular Hamiltonian in the adiabatic representation. For a CI to occur, the two eigenvalues of *Ĥ*_el_ must be equal (*E*_2_ = *E*_1_). In the two state case, it is possible to define an exact transformation from the adiabatic representation to the diabatic representation.^13–16,20^ The diabatic electronic wave functions (*ϕ*^el^_1_(*r*;*R*), *ϕ*^el^_2_(*r*;*R*)) can be determined as a linear combination of the adiabatic electronic wave functions:8
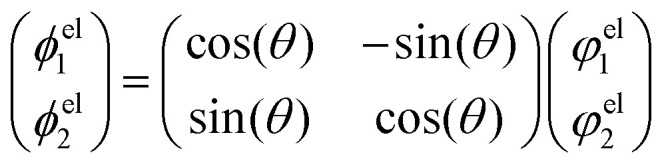
The mixing angle *θ* between the adiabatic and diabatic basis can be defined in the interval [0,π] and is obtained by requiring the nonadiabatic coupling elements *τ*_12_ to vanish. Following *θ* along a closed path of nuclear configurations that encircle CI, a phase shift of π is observed. This phase shift is called geometric phase^17,18^ and leads to a sign change of adiabatic electronic wave functions, if a closed path around the CI is followed. The diabatic electronic wave functions have the advantage that the diabatic coupling between the two electronic states is described by the potential-like scalar quantity *D*_12_ = 〈*ϕ*^el^_1_|*Ĥ*_el_*ϕ*^el^_2_〉, in contrast to the vector *τ*_12_ in the adiabatic representation. The corresponding bare molecular Hamiltonian ***H***^D^_M_ has the following form:9
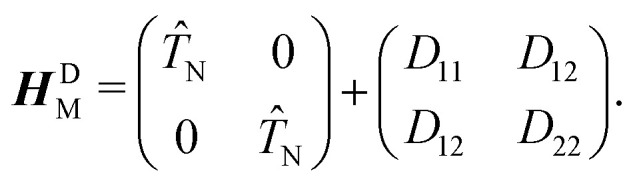
where *D*_11_ and *D*_22_ are the diabatic electronic energies. The two eigenvalues of *Ĥ*_el_ can be expressed in terms of diabatic energies and the diabatic coupling:10
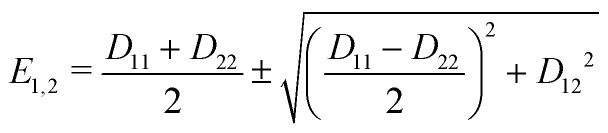
For a CI to occur, *E*_2_ and *E*_1_ must be equal, which is only possible if the following two conditions are fulfilled simultaneously:11
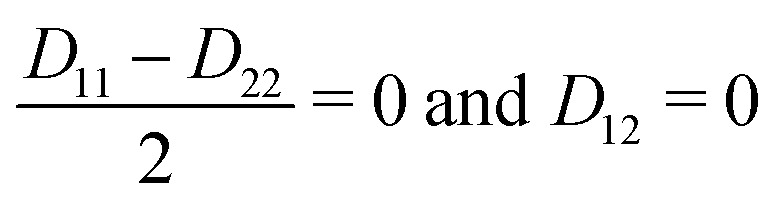
If *N* is the number of internal degrees of freedom of a molecule, each of the two conditions form an (*N* − 1)-dimensional subspace (for real-valued electronic wave functions). Their intersection describes a (*N* − 2)-dimensional space in which the electronic states are degenerate, also called intersection space. The remaining two-dimensional subspace is called branching space and defined by the gradient difference vector (*X*_1_) and the derivative coupling vector (*X*_2_). A schematic representation of a CI in the adiabatic representation is given in Fig. 1. The shown characteristic cone shape is only present in the adiabatic basis. Nevertheless, the CI itself can be defined independently of the representation used, and the induced strong electron–nuclear coupling in its vicinity is basis independent. In the case of diatomic molecules, there is only one internal degree of freedom. Therefore, only one of the two conditions (eqn (11)) can be fulfilled simultaneously, and a CI can not exist. If the involved states share the same symmetry, an avoided crossing is formed in the adiabatic representation and nonadiabatic processes can occur. For the case of different symmetries, the PESs can intersect, but no nonadiabatic coupling is present. This non-crossing rule,^13–16,65^ which is based on symmetry considerations, is strictly only valid in the diatomic case. For larger molecules, states of the same symmetry can form a CI and, in general, symmetry is not a rigorous constraint to form a CI in polyatomic molecules with more than one nuclear degree of freedom.

In the two-state situation, the adiabatic representation and the diabatic representation are formally equivalent, but this is no longer true for systems with more coupled electronic states. In such a situation, the diabatization can only be done in an approximate fashion leading to a quasi-diabatic or pseudo-diabatic representation.^66,67^

### B. Light–matter interaction

To start the excited states dynamics and to probe the nonadiabatic processes, a pump–probe scheme is used. We treat the pump-pulse explicitly and the interaction with the probe-pulse by means of time-dependent perturbation theory. The Hamiltonian describing the photoexcitation and the subsequent dynamics has the following form:

12 *Ĥ*_I_ = *Ĥ*_M_ + *Ĥ*_P_

*Ĥ*_M_ is the bare-molecule Hamiltonian and is formulated in the adiabatic or diabatic representation, as defined in eqn (7) and eqn (9). The light–matter interaction Hamiltonian *Ĥ*_P_ for the pump-pulse can be written under the rotating wave approximation (RWA) as follows,13
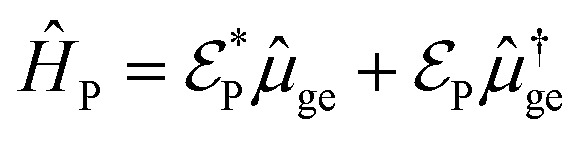
where *μ*_ge_ is the adiabatic or diabatic transition dipole operator between the ground and excited state, and 
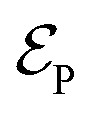
 is the electric field corresponding to the pump-pulse.14
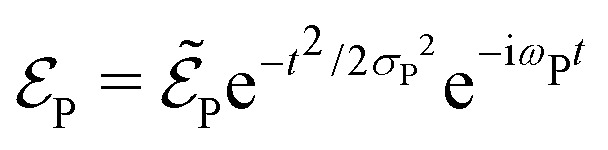
with 
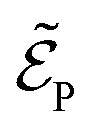
 being the electric field amplitude, *σ*_P_ being the pulse width, and *ω*_P_ being the center-frequency of the pump-pulse.

For the construction of pump–probe signals, the probe-pulse interaction is treated as a time-dependent perturbation to the photoexcited molecular Hamiltonian (eqn (12)). The resulting total Hamiltonian for the simulation of the pump–probe spectrum takes the form,

15 *Ĥ*(*t*) = *Ĥ*_I_ + *Ĥ*^(*μ*/*α*)^_X_(*t*)

where *Ĥ*_I_ is the Hamiltonian of the molecule prepared by the pump-pulse interaction (see eqn (12)), and *Ĥ*^(*μ*/*α*)^_X_ is the interaction Hamiltonian corresponding to the probe-pulse interaction, with *μ* and *α* in the superscript representing either resonant or off-resonant nature of the probe process. Interaction Hamiltonians for resonant and off-resonant probes can be written as,16
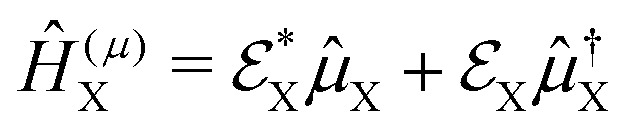
17
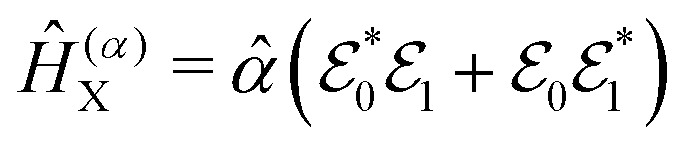
where 
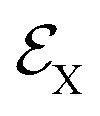
 and *μ̂*_X_ in eqn (16) represent the electric field for the probe-pulse and the corresponding transition dipole operator, respectively. In eqn (17), 
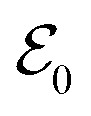
 and 
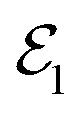
 represent two probe-pulses, and *α̂* represents the polarizability operator.

When the probe-pulse photon modes are distinct from the detection modes, which is the case in spontaneous emission spectroscopy, the total Hamiltonian reads,

18 *Ĥ*(*t*) = *Ĥ*_I_ + *Ĥ*^(*μ*/*α*)^_X_(*t*) + *Ĥ*_D_(*t*)

where *Ĥ*_D_ is the interaction Hamiltonian corresponding to the detection mode, and takes a similar form to the Hamiltonian in eqn (16).

The interaction of a molecule with the detection modes (given by *Ĥ*^(*μ*/*α*)^_X_ in eqn (15) and *Ĥ*_D_ in eqn (18) is treated as a perturbation. The signal may be defined as an integrated rate of change of photons in the detection mode, and can be written as,^68^19
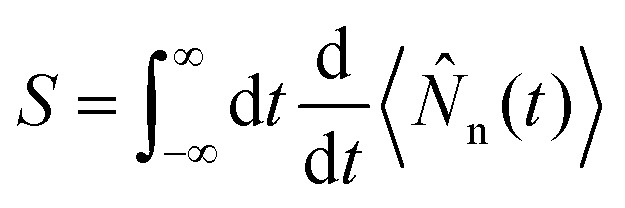
where *N̂*_n_ = *â*^†^_n_*â*_n_ represents the photon number operator with *â*_n_ (*â*^†^_n_) being the photon annihilation (creation) operator for mode n, and 〈*N̂*_n_(*t*)〉 = 〈*ψ*(*t*)|*N̂*_n_|*ψ*(*t*)〉 with *ψ*(*t*) being the wave function of the system at time *t*. The wave function of the system, *ψ*(*t*), can be expanded in terms of the perturbation Hamiltonian (*Ĥ*_X_^*μ*/*α*^ in eqn (15) and *Ĥ*_D_ in eqn (18)) using a Dyson series.

## III. Time-resolved spectroscopy and diffraction

Over the last decades, a variety of laser sources that allow the generation of highly tunable laser pulses have been developed. For instance, modern FEL facilities and HHG sources give access to X-ray pulses with a temporal resolution on the order of femtoseconds and sub-femtoseconds, along with a spectral range of up to a few eVs. These pulses are interesting candidates for probing ultra-fast processes in molecules. In the following section, we present several spectroscopic techniques, as well as a diffraction technique, which use ultra-short X-ray pulses to study different aspects of nonadiabatic phenomena occurring near a CI. The first reviewed technique is TXAS, which is well established in theory and experiment. In addition, we will discuss resonant and off-resonant spectroscopic techniques, such as XSES, TRUECARS, TRPES, and diffraction-based techniques such as XRD. Proposed techniques, such as TRUECARS and XRD, have not yet been demonstrated experimentally for the detection of CIs. However, theory suggests that these techniques may provide new insights into nonadiabatic processes.

**Fig. 2:**
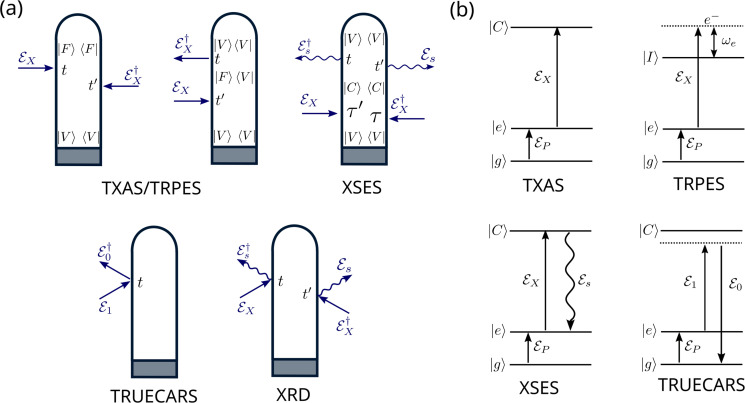
Overview over the diagrammatic representations for the spectroscopic techniques discussed in this feature article. (a) Loop diagrams for the presented signals. The grey box in each diagram, represents preparation process *via* the pump-pulse. TXAS and TRPES share two diagrams, which solely differ in the intermediate state |F〉: for TXAS |F〉 ≡ |*C*〉 is a core excited state, while for TRPES |F〉 ≡ |I〉 is an ionic state. In both cases, the system propagates in state |F〉 during the duration of the probe-pulse. In the diagram for XSES and XRD, straight arrows indicate the interaction with probe-pulse and the wiggled arrows indicate spontaneous emission. The TRUECARS diagram describes an off-resonant, stimulated Raman process. (b) Level diagrams for TXAS, TRPES, XSES, and TRUECARS are shown here. The pump-pulse 
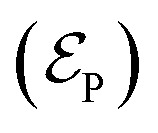
 interaction prepares the system to a non-stationary state that is probed using the probe-pulse, 
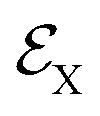
. States |g〉 and |e〉 represent ground and excited states, respectively. States |C〉 and |I〉 represent core-hole and ionic states, respectively.

A specific spectroscopic signal can be expressed through eqn (19) by using time-dependent perturbation theory. This allows for developing the signal expression in different orders with respect to the electric field. The result contains correlation functions, which depend on one or more interaction times, where the system interacts with the electric field. To keep track of the interactions, doubled sided Feynman diagrams,^68,69^ as they are shown in Fig. 2(a), are commonly used: time flows in the vertical direction and the left and right side represent the ‘ket’ and ‘bra’ of the correlation function, respectively. In and outgoing arrows represent an interaction with the electric field probing the system.

The first two diagrams, in Fig. 2(a), describe the correlation functions for TXAS as well as TRPES. The two interactions both originate from the probe-pulse. The major difference between the two techniques is that the state F represents a core excited state in the case of TXAS and an ionic state combined with a photoelectron in the case of TRPES. The corresponding level diagrams are shown in Fig. 2(b). Note that we use two different, but similar level diagrams to describe TXAS and TRPES, while we can use the same loop diagrams to describe both processes. The third diagram in Fig. 2(a) is a description of the spontaneous emission process (XSES). The two ingoing arrows describe the interactions with the X-ray probe-pulse and the outgoing arrows represent the emission of a photon. The corresponding level diagram is also shown in Fig. 2(b). The fourth diagram in Fig. 2(a) and (b) describes the off-resonant linear Raman process the makes up the TRUECARS technique. Note that both interactions here appear at the same point in time, and thus the interaction is mediated by the polarisability rather than a dipole interaction. The fifth diagram describes the time resolved XRD process. Note that there are two pairs of interactions here, each of which describes the off-resonant scattering of the probe-pulse on the charge density of the molecule. A common scheme between all presented diagrams here is that only signals are considered, which are linear in intensity with respect to the probe-pulse(s) (*i.e.* two interactions with the probe-pulse).

### A. Transient X-ray absorption spectroscopy

TXAS is a sensitive tool for following electronic and structural changes in molecules, which measures resonant transitions from element-specific core orbitals to valence orbitals. The direct probing of the valence orbitals allows monitoring of the bonding situation and active electronic states of the molecular system. If combined with ultra-short resonant pump-pulses, TXAS offers the opportunity to observe some of the fastest processes in photochemistry, including electronic state-switching dynamics at avoided crossings and CIs. If a resonant excitation is not possible in the experiment and non-resonant strong field pump-pulses are used, TXAS still provides detailed insights into the electronic-state-resolved dynamics.^70^ The valence-to-core excitation frequency changes as the nuclei evolve in an excited state PES. By varying the pump–probe delay, the information of the absorbed photons is used to follow the nuclear dynamics. If a CI is active in the molecule, the bifurcation of an absorption band can be expected to appear in the TXAS spectrum. This fingerprint of a CI could be observable under the condition that the population transfer is balanced and transition dipole moments corresponding to the relevant valence-to-core transitions have comparable strengths.

**Fig. 3:**
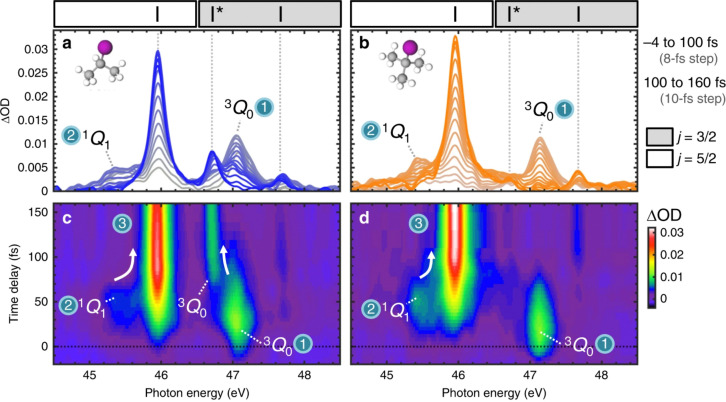
Experimental transient absorption spectra of iso-propyl iodide and *tert*-butyl iodide. (a and b) XUV-absorption spectra of iso-propyl iodide and *tert*-butyl iodide are plotted in grey colours that evolve to blue or orange increasing time delay. Dashed vertical lines indicate the positions of atomic iodine transitions. Regions of the spectra in which (4d_5/2_)^−1^ and (4d_3/2_)^−1^ core-level transitions appear are demarcated by white and grey boxes, respectively. Adjacent I (45.9 eV) and ^3^Q_0_ (47.1 eV) peaks overlap slightly in the 46.4–46.8 eV region. (c) and (d) Experimental transients for iso-propyl iodide and *tert*-butyl iodide plotted as colourmaps. State-specific molecular features and their convergence (indicated by arrows) to the atomic transitions are labelled. Reproduced from ref. 71. Creative Commons License CC BY 4.0.

An experimental example of this bifurcation being observed is the photo-initiated nonadiabatic fragmentation dynamics of different alkyl iodides.^71^ The dynamics are launched by a resonant UV pump-pulse and is followed by a time-delayed attosecond XUV pulse that probes transitions between iodine I(4d) core orbitals and valence orbitals of the dissociating molecules. The resulting experimental transient absorption spectra for iso-propyl iodide and *tert*-butyl iodide are shown in Fig. 3. The two electronic states involved ^3^Q_0_ and ^1^Q_1_ lead to distinguishable iodine fragments. The ^3^Q_0_ state forms mostly spin–orbit excited atomic I* (46.7 eV) and the ^1^Q_1_ state decays predominantly into ground state I (45.9 eV). After excitation to the ^3^Q_0_ the nuclear wave packet encounters an intersection region and branches into both states during the dissociation. The appearance of the iodine signal at 45.9 eV after 20 fs is a result of the bifurcation of the nuclear wave packet. Both systems show a similar behaviour and only differ in the branching ratio at the intersection.

Besides this example there are numerous experimental and theoretical studies in the literature discussing TXAS applied to the electronic structure change in molecules. In the following, we want to give a brief overview over some of these publications. Experimental efforts have been made towards observing the nonadiabatic dynamics present in the ring-opening reactions in 1,3-cyclohexadiene (CHD)^46^ and furfural^47^ using X-ray absorption spectroscopy. The ring-opening reactions in CHD and furfural following a UV excitation are believed to pass through two successive CIs. Recently, nonadiabatic dynamics in IBr were successfully observed using transient absorption with extreme ultraviolet (XUV) probes.^48^ The photoexcitation of IBr using a visible probe initiates a dissociation which passes through an avoided crossing. The passage of the molecule through the coupling region leads to the formation of two fragments upon dissociation, and is clearly visible in the absorption spectrum. In another experiment with transient absorption, the branching of wave packets near a CI is observed in methyl bromide.^49^ The electronic relaxation *via* a CI in ethylene had been experimentally observed using transient absorption with soft X-ray probes.^50^ Hints of a CI being active in silane has been recently observed in a transient absorption experiment studying electronic coherence between valence and Rydberg states.^52^ Based on high-level nonadiabatic dynamics simulations, the use of TXAS has been proposed to resolve competing CIs pathways in *trans*-1,3-butadiene.^72^

Within our simulation protocol, the studied molecule is excited using a resonant pump-pulse (see eqn (12)) and the signal expression for TXAS can be derived using eqn (19), and reads,^73^20
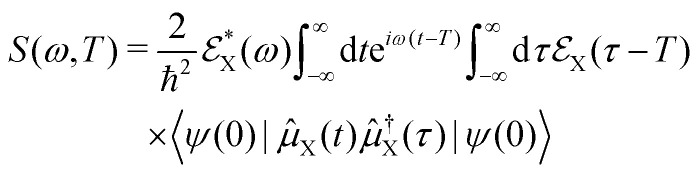
where *ψ*(0) is the wave function of the prepared molecule at time *t* = 0, 
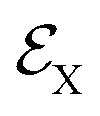
 is the electric field for the X-ray pulse, and *μ*_X_ is the transition dipole operator corresponding to the valence-to-core excitation. The corresponding loop diagram and the complementary state diagram are depicted in Fig. 2.

**Fig. 4:**
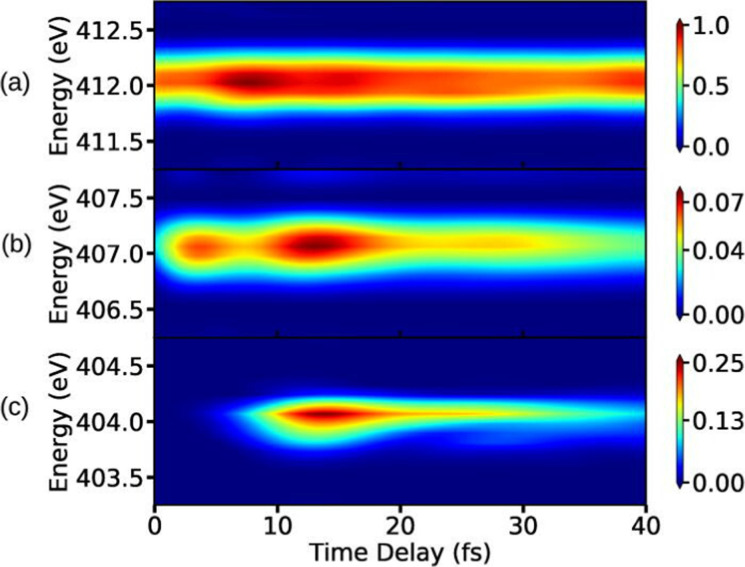
TXAS spectra simulated with eqn (20) for pyrrole obtained using different X-ray probe center frequencies *ω*_X_. (a) For *ω*_X_ = 412 eV, a signal with a nearly constant intensity appears, and it primarily stems from the ground state population. (b) For *ω*_X_ = 407 eV, the spectrum shows an overlap signal between Franck–Condon (FC) point to the e_1_ state contribution and the CI region to the e_2_ state contribution. (c) For *ω*_X_ = 404 eV, the signal appears when the wave packets reach the CI region. The pump-pulse and the X-ray probe-pulse have pulse widths of 1 fs and 1.5 fs, respectively. Reproduced from ref. 53. Copyright 2021 AIP Publishing, Creative Commons License CC BY 4.0.

To complete this section with a second illustrative example, we discuss the simulated TXAS spectra for pyrrole molecule.^53^ The UV-induced photochemistry of this nitrogen-containing five-membered heterocyclic aromatic compound is characterised by a NH detachment, and along this dissociation process, the ground state and the first excited state (πσ*) form a CI. For more details on the molecular system and the simulation protocol, we refer to ref. 53. The simulated TXAS spectra for the pyrrole molecule are shown in Fig. 4. The TXAS signal shown in Fig. 4(a) is constructed for an X-ray centre frequency of 412 eV, which is resonant to the ground state minimum to the first nitrogen 1s core-hole state transition, among other valence-to-core transitions. The spectrum shows a signal with nearly constant intensity throughout the probe delay, and it primarily stems from the ground state population that remains nearly constant after the pump process. The TXAS signal shown in Fig. 4(b) is constructed using an X-ray center frequency of 407 eV, and is an overlap of two signals primarily: (i) the signal originating from the transition between the first valence excited state and the first nitrogen 1s core-hole state (referred as e_1_ hereafter), and (ii) the signal from the transition between the CI region and the second nitrogen 1s core-hole state (referred as e_2_ hereafter). The signal intensity increases until the pump-pulse interacts with the molecule and then starts decreasing after ≈5 fs when the wave packets move away from the Franck–Condon (FC) region. The increase in intensity again around 10 fs is due to the overlap of the absorption signal corresponding to the CI region to e_2_ state transition. The spectrum shown in Fig. 4(c) corresponds to the CI region, and is constructed with an X-ray centre frequency of 404 eV, which is among the lowest transition frequencies in the considered model. The signal starts to build around 10 fs when the wave packets approach the CI region. The branching of the wave packets near the CI does not appear due to a small population transfer (≈7% of the πσ* state population) near the CI. However, the signal intensity in Fig. 4(c) is one order of magnitude stronger than the signal intensity in Fig. 4(b) despite it being an overlap of two signals. The increase in the intensity of the signal stems from a maximum in the transition dipole moment between the core-hole and valence states near the CI. This signature can be interpreted as a fingerprint of the CI which connects the valence states.

### B. Time-resolved X-ray spontaneous emission spectroscopy

After the absorption of an X-ray photon, the molecule is in a core-hole state, which has a lifetime on the order of a few femtoseconds.^74,75^ For lighter elements, the Auger–Meitner^76–78^ process can be the dominant decay channel but also the spontaneous emission back to the valence states contributes to the overall relaxation process. In principle, both the Auger–Meitner decay^77^ as well as the spontaneous emission can be used to study ultra-fast excited state dynamics in molecules. In this section, we discuss how the spontaneously emitted photons can be utilised to visualise the intersecting electronic states near a CI.

Since the spectroscopic signal is constructed by recording the spontaneously emitted photons, the preparation process of the molecule involves interaction with the pump-pulse initiating the valence states dynamics, and the probe-pulse populating the core-hole state. The XSES signal expression can be obtained using eqn (19) for spontaneous emission photons which work as detection modes. The time-resolved XSES signal expression reads,^79^21
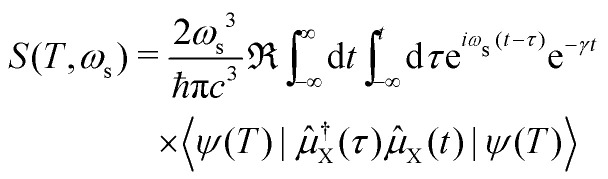
with *γ* being the decay rate for core-hole states, and |*ψ*(*T*)〉 is the wave function of the molecule prepared by the X-ray pulse with pump–probe delay of *T*. The signal is visualised as loop diagram and in form of a state diagram in Fig. 2.

**Fig. 5:**
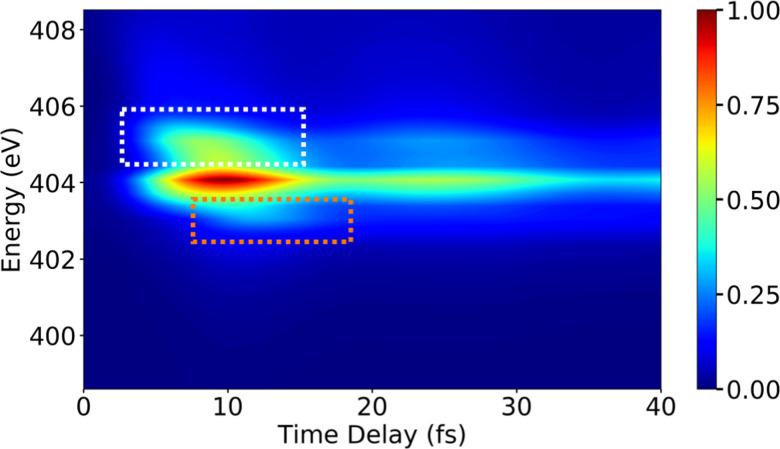
The XSES spectrum simulated with eqn (21) for pyrrole for the e_1_ state relaxation *via* spontaneous emission. Signals in white and orange dotted boxes correspond to the S_0_ electronic state before and after the passage through the CI, respectively. Whereas, the signal around 404 eV stems from relaxation to the πσ* state. The intersection of the electronic states near the CI is visible around 10 fs. The pump- and X-ray probe-pulse widths are 1 fs and 0.5 fs., respectively. Reproduced from ref. 53. Copyright 2021 AIP Publishing, Creative Commons License CC BY 4.0.

Following the X-ray excitation to a core-hole state, a molecule may relax to multiple valence states *via* spontaneous emission. The corresponding probability of the relaxation to a specific valence state can be approximated by Einstein's A-coefficient.^80^ Assuming the molecule of interest relaxes from a single core-hole state to two valence states, two peaks in the XSES spectrum appear, and the separation between the peaks gives the energetic separation between the two valence states. If these two states form a CI, the time-resolved XSES spectrum may show two peaks approaching each other before the CI and separating afterwards. For the illustration of the XSES technique we again consider the nonadiabatic dynamics of pyrrole.^53^ The time-resolved XSES spectrum is simulated using eqn (21), and is shown in Fig. 5.

Since we are interested in the dynamics in the vicinity of the CI between states S_0_ and πσ*, an X-ray pulse with a center frequency of 403.3 eV is used, which is in near-resonance to the separation between the CI region and the e_1_ state. The signal starts to build after ≈7 fs, and achieves the peak intensity around 11 fs. A signal appears throughout the time-delay at 404 eV, which corresponds to the spontaneous emission from the e_1_ state to the πσ* state. The persistence of the signal at 404 eV for over 30 fs is due to the similar shapes of the PESs for states e_1_ and πσ*. Alongside the main peak at 404 eV, there are two side bands (indicated by orange and white boxes) that appear in the spectrum. The signals in the white and orange boxes stem from the spontaneous emission from the e_1_ state to the S_0_ state before and after the CI, respectively. The signal from the S_0_ state appears above 405 eV at ≈7 fs and appears around 403 eV at ≈15 fs, whereas the signal from the πσ* state appears around 404 eV throughout. In the XSES spectrum, the separation between the two valence states can be seen changing between 5 fs and 15 fs, and their intersection is happening around 10 fs. Therefore, XSES spectra may provide additional information about a CI by visualisation of the evolving electronic state separation.

### C. TRUECARS

One of the prominent features of a CI is the creation of an electronic coherence when the nuclear wave packet branches near a CI. The time evolution of the generated electronic coherence carries information about the energetic separation between the two involved electronic states. Therefore, studying this electronic coherence may help deduce the changing electronic state separation after the passage through the CI. Multiple off-resonant stimulated Raman methods have been proposed to study coherence present in a molecule.^81–84^ In a stimulated Raman process, two temporally overlapping pulses are used as a probe that creates a Raman transition between two electronic states. One of these stimulated Raman spectroscopic methods is TRUECARS,^51^ which detects the occurrence of CI by relying on the generated electronic coherence near a CI.

**Fig. 6:**
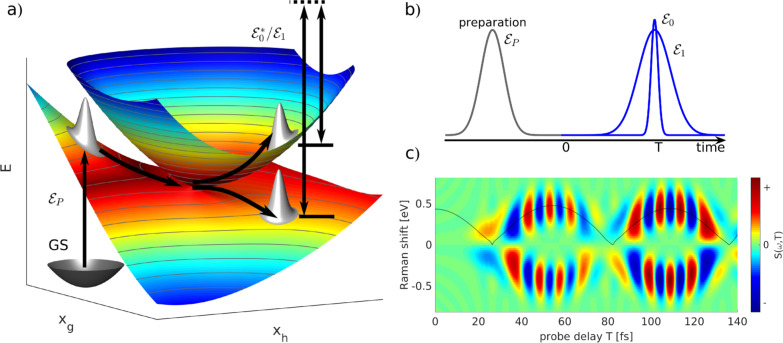
A schematic representation of the TRUECARS technique. (a) Adiabatic potential energy surfaces for the electronic states with a CI are shown. A pump-pulse 
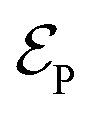
 creates an excitation from the ground state to an excited state. The nuclear wave-packet evolves on the energy surfaces until it reaches the coupling region. When the nuclear wave packet goes through the coupling region, an electronic coherence is generated between the involved electronic states. The created electronic coherence is probed by using the Raman probe with a broadband 
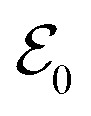
 and narrowband 
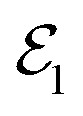
 hybrid pulse scheme. (b) A pulse scheme for the pump- and hybrid probe-pulses. (c) A TRUECARS spectrum for a 1D model system. The Raman shift appears along the vertical axis and the spectrum follows the black line, which represents the electronic state separation curve between the states with the electronic coherence. Reproduced with permission from ref. 51. Copyright 2015 American Physical Society.

Within the TRUECARS schema, a molecule is probed by the interaction with a combination of two overlapping X-ray pulses. The two X-ray probes, 
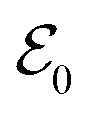
 and 
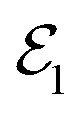
, can constitute a hybrid pulse scheme, in which 
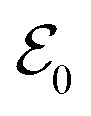
 can be considered to be a broadband pulse and 
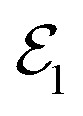
 can be considered to be a narrowband probe, as shown in Fig. 6(b). However, the use of a broadband-narrowband hybrid probe is not a necessity, and it is possible to use both probes with the same pulse widths. The signal may be defined as an integrated rate of change of photons of 
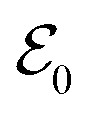
 field, and the signal expression reads,^51^22
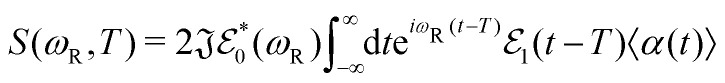
where *ω*_R_ is the Raman frequency, *T* is the pump–probe delay. The corresponding diagrams for the TRUECARS signal can be found in Fig. 2. The polarizability tensor *α* has the following form:23
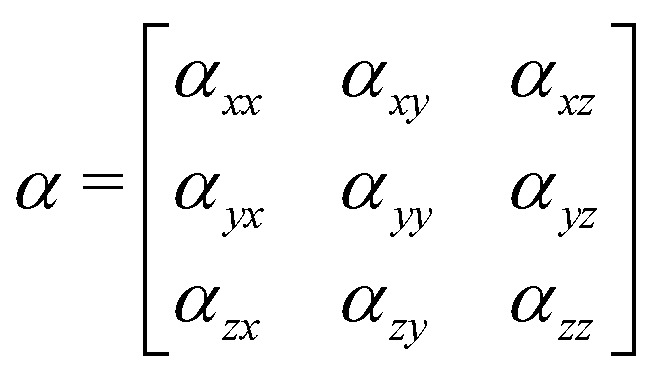
where each element *α*_*ij*_, with *i*, *j* = *x*, *y*, *z*, of the polarizability tensor gives directions of the polarization. The tensor elements are expanded in the basis of the valence states of the system and can be written as:24
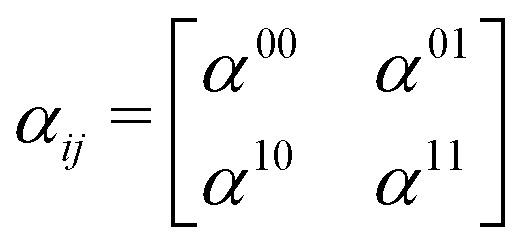
where each matrix element *α*^kn^, with k, n being the initial and final states, depends on the frequency of the probe-pulses *ω* along with the nuclear coordinates of the system, and can be written as:^85^25
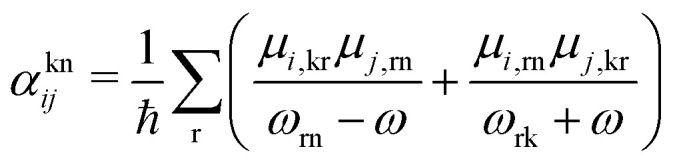
where *ω*_rn_ = (*E*_r_ − *E*_n_)/*ħ* with *E*_n_ and *E*_r_ being the energies of the valence state n and the core-hole state r, respectively.

The use of eqn (25) in eqn (24) leads to the creation of a non-Hermitian matrix. Therefore, under the assumption that *ω*_rn_ − *ω* is much larger compared to the energy difference between the valence states, the off-diagonal elements (*α*^01^ and *α*^10^) in eqn (24) can be written as the average: *α*′ = (*α*^01^ + *α*^10^)/2. This leads to a new polarizability matrix that is Hermitian in nature,26
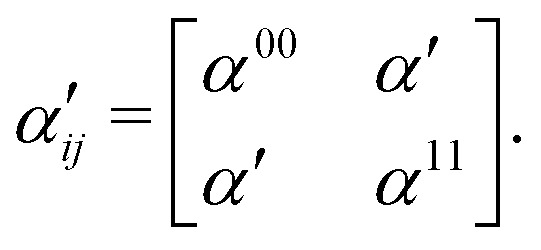


Assuming that the diagonal elements in eqn (26) are constant, the TRUECARS spectrum shows only features corresponding to the electronic coherences generated by CI, and the changing electronic state separation can be observed, as shown in Fig. 6(c). In general, this is not the case in molecules, and both diagonal and off-diagonal elements of the polarizability matrix need to be considered.

**Fig. 7:**
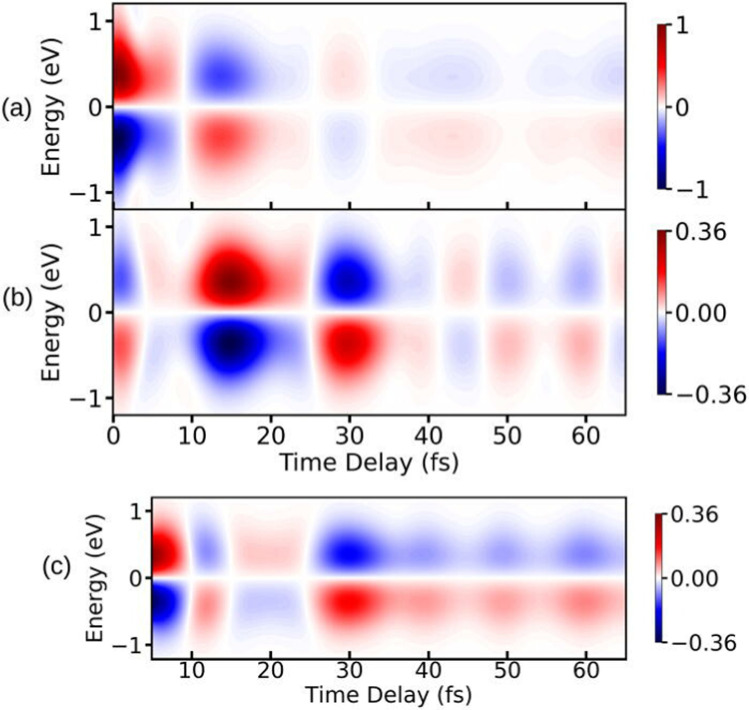
TRUECARS spectra simulated using eqn (22) for different polarizability tensor elements in pyrrole. (a) The signal for *α*_*xx*_ is strong before the passage through the CI and decays after 10 fs. (b) The signal for *α*_*yy*_ is weak before 10 fs and builds up during the passage through the CI. (c) The total (*α*_*xx*_ + *α*_*yy*_) signal has a vanishing intensity between 10 fs and 20 fs due to the opposite phases of signals in (a) and (b). Hybrid probe-pulses, *ε*_0_ and *ε*_1_, have pulse widths of 0.9 fs and 1.7 fs, respectively. Reproduced from ref. 53. Copyright 2021 AIP Publishing, Creative Commons License CC BY 4.0.

To illustrate the properties of the TRUECARS detection schema, we again turn to the photo-dissociation of pyrrole.^53^ The TRUECARS spectra constructed for pyrrole are shown in Fig. 7. The signal contributions for the non-zero diagonal components of the polarizability tensor component are shown in Fig. 7(a)–(c). The signal generated by *α*_*xx*_ (Fig. 7(a)) is stronger before the CI (<10 fs), while the signal generated by *α*_*yy*_ (Fig. 7(b)) is stronger during and after the passage through the CI (≥10 fs). Due to the opposite phases of the signals from *α*_*xx*_ and *α*_*yy*_, the total signal has a reduced intensity while the system goes through the CI. Therefore, the occurrence of the CI can be detected in the total spectrum due to the vanishing signal between 10 fs and 30 fs.

In the last few years, extensions for the TRUECARS technique have been proposed, which improve the selective with respect to electronic coherence and may help to realise the method experimentally.

**Fig. 8:**
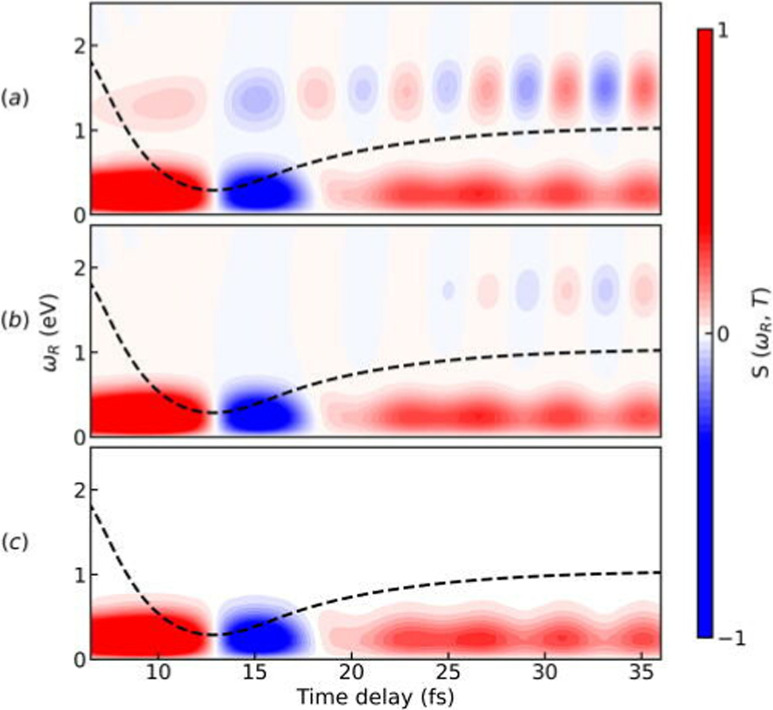
Simulated TRUECARS spectra using eqn (22) where an attosecond pulse train (APT) is used as _0_ pulse and a Gaussian pulse is used as _1_ pulse. The seed pulse frequencies that are used to create the attosecond pulse train (APT) are (a) *ω*_I_ = 0.83; (b) *ω*_I_ = 0.99; (c) *ω*_I_ = 1.55 eV. The Gaussian pulse employed as *ε*_1_ has a width of *σ*_1_ = 1.5 fs. The dashed black line represents the average time-dependent separation of the adiabatic potential energy surfaces. Reproduced from ref. 86. Copyright 2022 AIP Publishing, Creative Commons License CC BY 4.0.

The combination of attosecond pulse trains (APTs) as the 
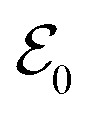
 pulse and a Gaussian pulse as the 
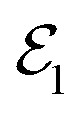
 pulse has been applied to improve the visibility of the electronic coherence overshadowed by vibrational components of the TRUECARS signal.^86^ Another benefit of APTs is that single attosecond pulses (SAPs) are no longer required as probes, which need to be filtered from the HHG output. Fig. 8 shows the TRUECARS spectra calculated for a model system with an APT as 
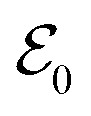
 and a Gaussian pulse as 
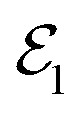
 with different seed pulse frequencies. Using an APT as 
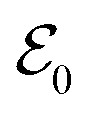
 produces a comb-like structure in the spectral domain and allows the placement of nodes on convenient energies. In Fig. 8(a), slowly oscillating Raman features, which correspond to the vibrational coherence in the system, appear below 1 eV, and oscillations corresponding to the electronic coherence appear above 1 eV. When the seed pulse frequency is increased to 0.99 eV (Fig. 8(b)) and 1.55 eV (Fig. 8(c)), features from the electronic coherence in the TRUECARS signal start to fade away since the node of the frequency comb hides the electronic coherence. Recently, another modification to the TRUECARS schema was proposed^87^ that enables the usage of stochastic X-ray pulses from current FEL sources. By taking advantage of the correlations of the field, this stochastic TRUECARS can provide the same temporal and spectral resolution as standard TRUECARS using phase-controlled pulses.

Although not yet experimentally realised, TRUECARS spectra have already been simulated for several molecular systems with interesting nonadiabatic processes. For example, the photo-relaxation *via* CIs in biological relevant molecules like uracil^24^ and its deviate 2-thiouracil^88^ were studied. The simulated TRUECARS spectra predict CI-induced coherences which live for hundreds of femtoseconds. The TRUECARS method was also applied to the nonadiabatic dynamics of highly branched multichromophoric conjugated macromolecules after photoexcitation.^83^

### D. Time-resolved photoelectron spectroscopy

Photoelectron spectroscopy is a mature technique that has been used extensively to study the electronic structure of molecules, solids, and atoms. Multiple variants of this technique have emerged over the years, for example, TRPES,^89^ angle-resolved photoelectron spectroscopy (ARPES),^90^ X-ray photoelectron spectroscopy (XPS).^91^ With the emergence of attosecond physics, photoelectron spectroscopy has been used to characterise ultrashort laser pulses rather than to study matter.^92^

By interacting with a suitable light pulse, a molecule, or an atom can be ionised, and the released photoelectron carries information about its parent ion. Our focus here is on TRPES, in which the ionising pulse interacts with the molecule with a varying pump–probe delay. TRPES has been used to study non-stationary process taking place on an ultrashort timescale, thus providing stroboscopic visualisation of such processes. Multiple theoretical studies using TRPES have been performed to investigate nonadiabatic dynamics in a molecular system.^93–100^ The ultra-fast nonadiabatic dynamics in NO_2_ has been experimentally studied using TRPES.^101^ The construction of an angular distribution with TRPES has been proposed to study nuclear dynamics in the vicinity of a CI.^102^

The use of TRPES to observe electronic coherence generated near a CI has been proposed in ref. 57. A full-quantum mechanical treatment of the photoelectron process leads to the appearance of quantum coherent oscillations in the simulated TRPES signal, which are generated by the electronic coherence. The signal may be defined as the integrated photoelectron current using eqn (19). The resulting signal expression reads,27
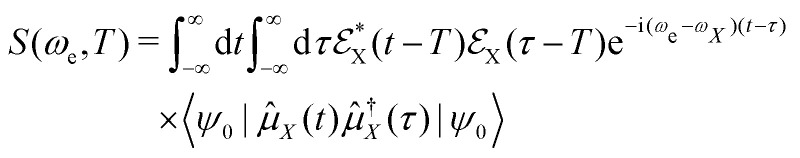
where *ω*_e_ is the photoelectron energy, 
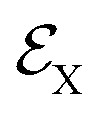
 is the electric field for the ionisation pulse with *ω*_X_ being the centre frequency of the pulse, *T* is the pump–probe delay, *μ̂*_X_ is the transition dipole operator corresponding to the ionisation process and *ψ*_0_ is the initial wave function of the prepared system. The TRPES signal expression can be read off a loop diagram, and the underlying process is visualised as a state diagram in Fig. 2.

**Fig. 9:**
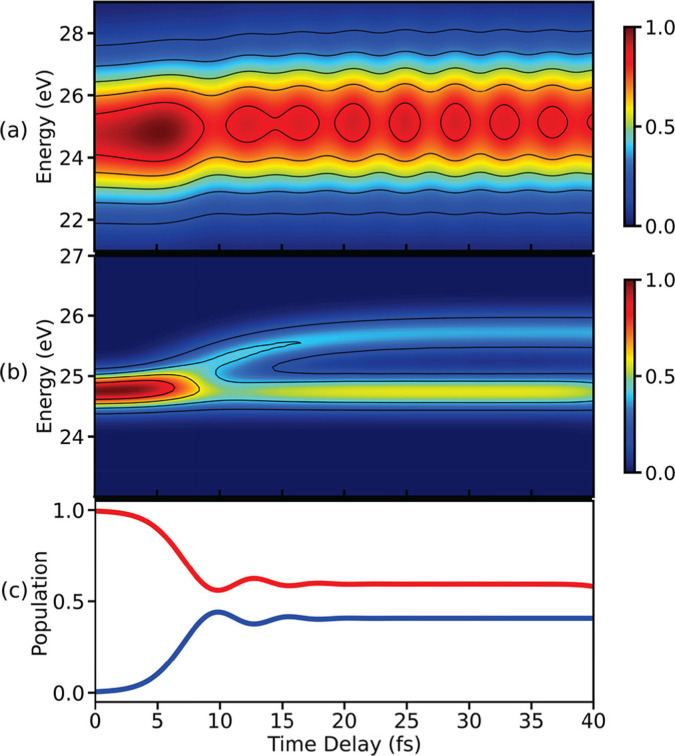
TRPES spectra simulated using eqn (27) for single ionisation pulses with pulse widths of (a) 0.3 fs and (b) 2.25 fs. The use of an ultrashort probe leads to the appearance of a beating pattern in (a) due to the electronic coherence created in the molecule *via* the CI. The few-femtosecond pulse used in (b) resolves the branching of the wave packets. (c) The temporal evolution of the population in states V_0_ (blue) and V_1_ (red) visualising the CI induced dynamics in a model system. Reproduced with permission from ref. 59. Copyright 2021 American Chemical Society, Creative Commons License CC BY 4.0.

TRPES signals for a model system with a CI are simulated using eqn (27), and are shown in Fig. 9(a) and (b). The signal in Fig. 9(a) is obtained using an ionisation pulse with a pulse width of 0.71 fs. As can be seen in Fig. 9(a), quantum beats appear in the signal after the passage through the CI after 10 fs, as evident from the population dynamics shown in Fig. 9(c). However, the branching of the wave packets near a CI is not visible in Fig. 9(a) due to poor spectral resolution. The spectral resolution in the photoelectron signal can be improved by using a longer ionisation pulse, as shown in Fig. 9(b). The branching of wave packets after 10 fs can be clearly seen. However, the quantum beats due to the electronic coherence do not appear anymore in the signal. The oscillations appear only when the photoelectron bands generated from the two valence states overlap with each other. Therefore, the branching of the wave packets and the oscillations due to the electronic coherence cannot be observed simultaneously in the TRPES signal using isolated probe-pulses. This restriction stem from the fact that TRPES is linear with respect to probe field. It allows for only one time variable in the spectrum, and is thus Fourier limited. However, this restriction can be lifted by introducing a second time variable.

One possibility of adding another time variable is to use attosecond streaking.^58^ In attosecond streaking, the photoelectron generated using an XUV pulse is exposed to an intense infrared (IR) field. When the photoelectrons interact with the IR pulse, sidebands are created. Their spacing corresponds to the IR carrier frequency. The delay of the IR field can be regarded as a second time variable, which allows for an increase in time resolution together with the energy resolution.^58^ The electronic coherence in the system can then be recovered by creating overlapping photoelectron bands, which leads to a characteristic oscillation pattern in the spectrum.

A different method suggested^59^ to partially bypass the Fourier restriction on the photoelectron signal is the use of an APT as the ionisation pulse. The peaks in the frequency comb of an APT in spectral domain can be denoted as odd harmonics of the seed pulse, which is used in the process of HHG to generate an APT. Ionisation using an APT with *n* peaks in the spectral domain creates *n* photoelectron peaks that mimic the comb-like structure in the spectral domain. For a two-level system, 2*n* photoelectron peaks would be generated when ionized using an APT. If the energy separation between the harmonics of the APT resonates with the separation between the two states in the two-level system, multiple photoelectron peaks would overlap. If the two-level system has an already existing electronic coherence, the overlap between the photoelectron peaks makes the quantum beats visible.

**Fig. 10:**
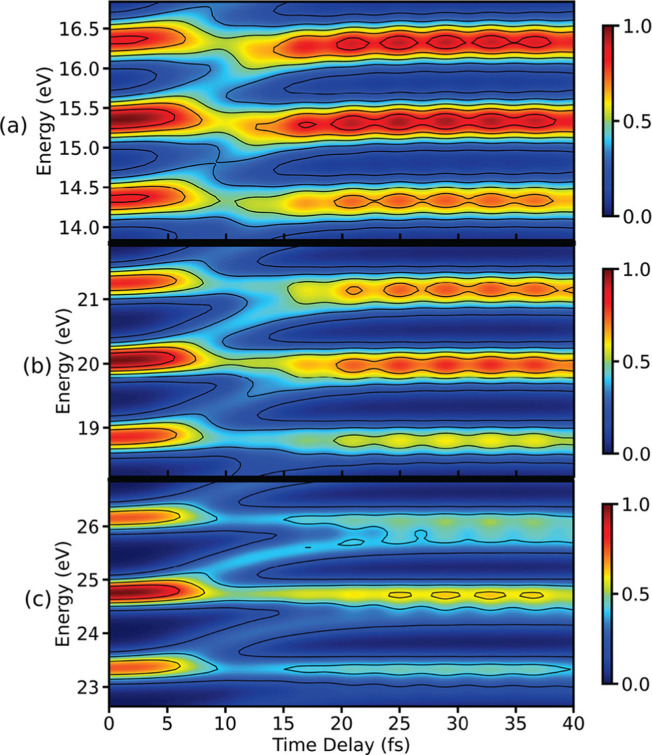
TRPES signals simulated using eqn (27) for an APT as an ionisation pulse are shown here. The seed pulse frequencies used for the generation of the APT are (a) *ω*_I_ = 0.5 eV, (b) *ω*_I_ = 0.6 eV, and (c) *ω*_I_ = 0.7 eV. For all three seed pulse frequencies, the branching of the wave packets and the coherent oscillations caused by the electronic coherence is observed. The APT has a pulse width of 2.55 fs where each attosecond pulse in it has a pulse width of 0.25 fs. For the demonstration purpose, only the three strongest peaks are shown in each spectrum. Reproduced from ref. 59. Copyright 2021 American Chemical Society, Creative Commons License CC BY 4.0.

TRPES signals constructed with an APT as an ionisation pulse are shown in Fig. 10 for different seed pulse frequencies. Note, that the model system used in the construction of the TRPES signal in Fig. 9 is used here. In Fig. 10(a), three main peaks appear until 10 fs and the branching of the wave packets can be seen after that. When the branched photoelectron peaks overlap with the main peaks, oscillations due to the electronic coherence appear. The strength of the quantum beats depends on the resonance between the seed pulse frequency and the electronic state separation.

### E. X-Ray diffraction

X-ray diffraction is commonly used to determine the structure of molecular samples and crystals. With the introduction of hard X-ray FELs, time-resolved diffraction experiments, which allow for a time-resolved tracking of molecular geometries, have become possible.^88,103–105^ In an experiment involving gas-phase electron diffraction of CH_3_I, nonadiabatic dynamics in a Rydberg state could be tracked in real-space.^106^ The electron diffraction signal clearly showed the bifurcation of the nuclear wave packet from the populated Rydberg state to an ion-pair state. In recent theoretical studies, X-ray and electron diffraction were used to study nuclear dynamics in the vicinity of CIs.^55,56,107^ The use of a resonant infrared (IR) pulse combined with an X-ray diffraction pulse has been recently proposed to enhance the features of coherences in the vicinity of a CI.^108^ Recently, a time-resolved orbital angular momentum X-ray pulse diffraction technique was proposed.^109^ The use of the twisted X-ray pulses that carry a light orbital angular momentum in theory allow the direct measurement of transient electronic coherences in gas-phase molecules, since the contribution of electronic populations to this signal is cancelled out.

Here, we focus on the single particle X-ray diffraction signal that depends on charge densities as well as transition charge densities and should, in principle, contain contributions which are related to electronic coherences. Scattering of an off-resonant X-ray photon gives rise to the diffraction signal,^110^ and is solely dependent on the charge density. The X-ray diffraction signal may be defined as an integrated rate of change of photons (see eqn (19)) of the scattered photon mode. The signal expression is derived using time-dependent perturbation theory, considering the minimal coupling Hamiltonian. The expression for the single particle diffraction signal then reads:^54^28
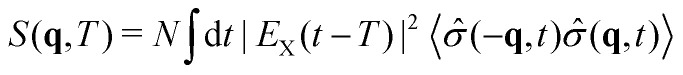
where *N* is the number of molecules in the disordered sample, *E*_X_(*t* − *T*) is the X-ray envelope with pump–probe delay *T*, **q** is the momentum transfer vector, *σ̂* is the charge density operator, and the expectation value 〈⋯〉 is taken over both electronic and nuclear degrees of freedom. The loop diagram, shown in Fig. 2, has two interactions with the probe-pulse, and thus represents a linear signal. The time-resolved X-ray diffraction signal that depends on nuclear coordinates takes the following form,29
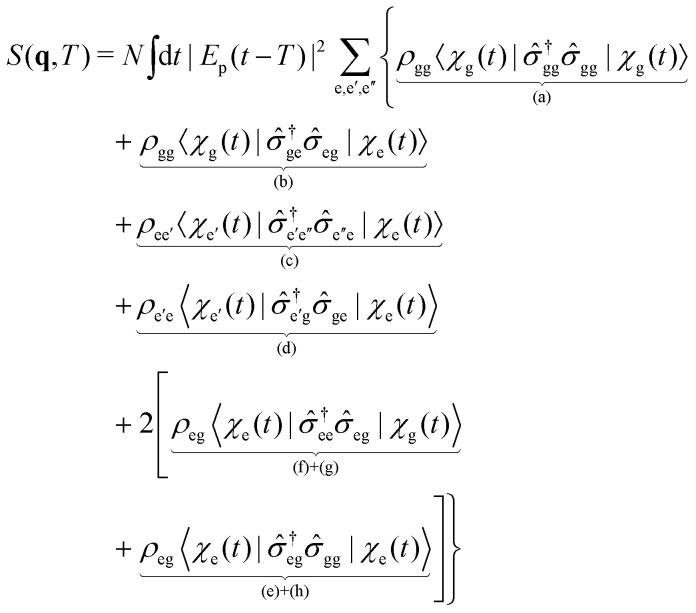
where e, e′, e′′ are electronically excited states, *ρ*_ii_ (i = e,g) are the electronic state populations, and the **q**-dependence is omitted for brevity. The first four terms (a), (b), (c), (d), represent the elastic and inelastic scattering contributions from the ground state, and the excited state. The latter terms (e), (f), (g), and (h) contain inelastic process, which scatter off electronic coherences, and contain information on the CI.

**Fig. 11:**
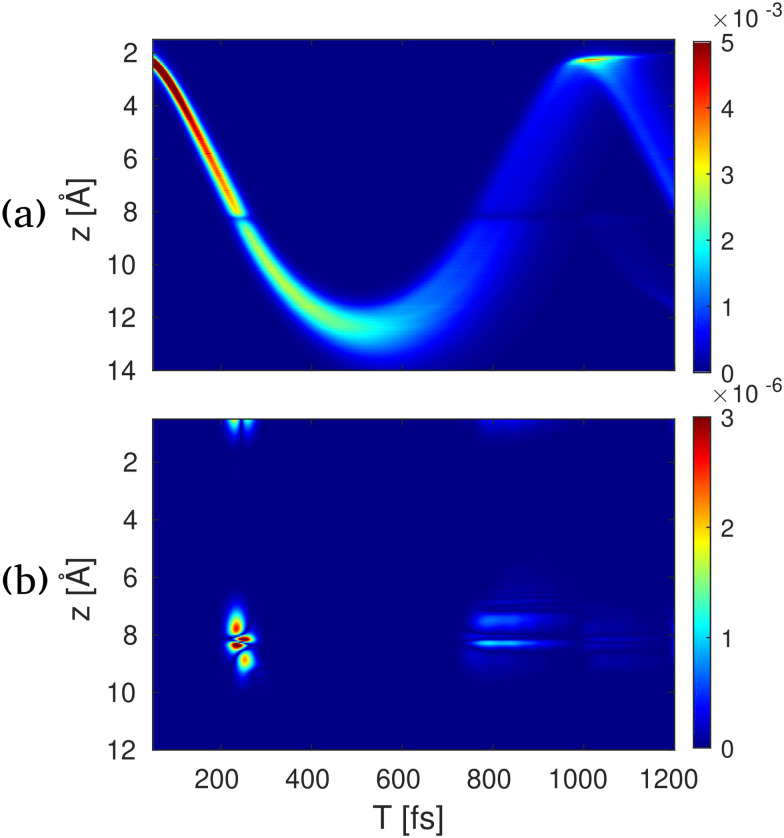
Time-resolved X-ray diffraction spectra for NaF. (a) The real space signal contribution to *S*_1_(*z*,*T*) calculated by the inverse Fourier transform of *S*(*q*_*z*_,*T*) in eqn (28) for elastic excited to excited-state contribution, (b) electronic coherence contribution between the ground and excited states. The bond length in NaF is shown along the vertical axes, and the pump–probe delay is shown along the horizontal axes. Reproduced with permission from ref. 54. Copyright 2018 PNAS.

A simulation of eqn (29) for the NaF molecule in gas phase is shown in Fig. 11. Here, a 10 fs UV pump-pulse launches the nuclear dynamics in the electronic A^1^Σ state. The probe-pulse is assumed to be a 2.5 fs long hard X-ray pulse. Fig. 11(a), shows the real-space representation of the elastic scattering contributions from the excited state, which directly corresponds to the probability density of the nuclear wave packet. Here, the back and forth oscillation of the nuclear wave packet can be clearly observed. In Fig. 11(b) the real-space representations of the relevant inelastic scattering contributions are shown. At around 200 fs the molecule passes through the avoided crossing between the A^1^Σ and the X^1^Σ state. A short-lived electronic coherence between the involved states is created. At this point in time, one can observe a diffraction image located at around 8 Å, which coincides with the location of the avoided crossing. In contrast to the elastic contributions, the shape of this feature is determined by the electronic transition charge density, rather than the electron charge density. The shape of the transition charge density can readily be approximated by the product of the highest occupied molecular orbital and the lowest unoccupied molecule orbital.

## IV. Potential future fields of study

So far, we have presented spectroscopic methods that can be used to study the molecular dynamics in vicinity of a CI formed by two electronic states. In the following, we discuss two scenarios that have not been investigated extensively in the context of ultra-fast spectroscopy. The first scenario are three-state CI, which have only been studied theoretically.^111–114^ Here, three electronic states become degenerate to form a CI in a 5-dimensional branching space (instead of a 2-dimensional branching space).^111^ The second scenario discusses light-induced conical intersections (LICIs) as an alternative to intrinsic CIs. Here, an external electromagnetic field is used to create a CI between dressed states.

### A. 3-State conical intersection

A three-state CI can be characterised by the simultaneous branching of the nuclear wave packet into three electronic states. However, this event may not be easily distinguished from a 2-state CI by means of tracking the electronic state populations with, for example, TXAS. A technique, such as XSES, may facilitate the detection of three emerging electronic states in the energy domain. However, a more unique signature would be the emergence of three distinct electronic coherences, while the wave packet passes through the 3-state CI. Each pair of electronic states involved, would carry a distinct frequency, which corresponds to the energy between the pair states.

Here, we demonstrate how the TRUECARS technique could be used to detect a three-state CI. We use a simplified model system, which involves three linear potential energy curves that intersect in a common point. The dynamics of the nuclei is treated classically:30
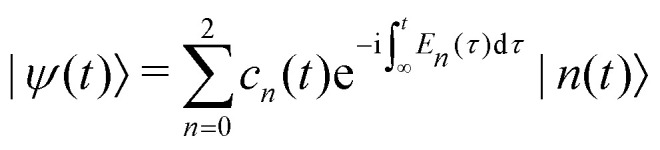
where *n* is the electronic state index with time-dependent energy *E*_*n*_(*t*). The TRUECARS spectrum can then be constructed using eqn (22) for constant polarizabilities with the wave function from eqn (30).^51^

**Fig. 12:**
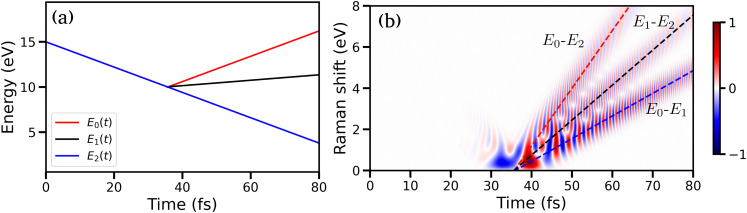
Model system for a 3-state CI: (a) semi-classical energy curves *vs.* time for the three electronic states involved in the three-state CI. (b) Corresponding TRUECARS spectrum for the model system. The signal appears after 30 fs at 0 eV Raman shifts, and then branches into three bands that correspond to three distinct sets of electronic coherence generated. The energy separation between the two electronic state in each set of electronic coherence can be tracked by corresponding bands in the spectrum. Pulse widths for the probe-pulses 
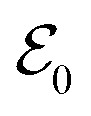
 and 
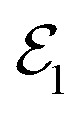
 are 0.5 fs and 2 fs, respectively.

The semi-classical time-dependent energy curves for three electronic states are shown in Fig. 12(a) (details of the model can be found in Appendix). Initially, one state is populated, and one curve appears until the system reaches the CI at 36 fs. After the passage through the CI, two more states are populated, and three electronic coherences are generated between all three pairs of electronic states. The resulting TRUECARS spectrum is shown in Fig. 12(b). The signal builds up around 30 fs when the wave packets branches from one state to the other two states. After ≈50 fs, three distinct bands can be observed in the spectrum. Each band originates from a pair of states and represents the energy separation between the states involved: the red, black and blue-dashed curves in Fig. 12(b) show energy separation between state pairs {0,2}, {1,2}, and {0,1}, respectively. The signal intensity decays over time as the separation between the electronic states increases and the wave packet overlap decays. Note, that the oscillation pattern in each of the bands carries the phase information between the respective states. Even though the model presented in Fig. 12 is strongly simplified, it shows that TRUECARS has the potential to unambiguously identify 3-state CIs.

### B. Light-induced conical intersections

As discussed in the theory section, the formation of a CI requires at least two independent nuclear degrees of freedom. In the presence of intense laser fields or a quantized vacuum field, a molecule can form dressed states, which results in a new set of states. Here, the electromagnetic field is an additional degree of freedom, and the orientation between the molecular transition dipole and the electric field can be used to create a degenerate point.^115,116^ As a result, a CI can appear in diatomics when they are exposed to resonant light field fields. The resulting CIs are referred to as LICIs. Several studies have emphasised the strong impact of LICIs on molecular spectra and quantum dynamics, even with weak laser fields.^116–123^

**Fig. 13:**
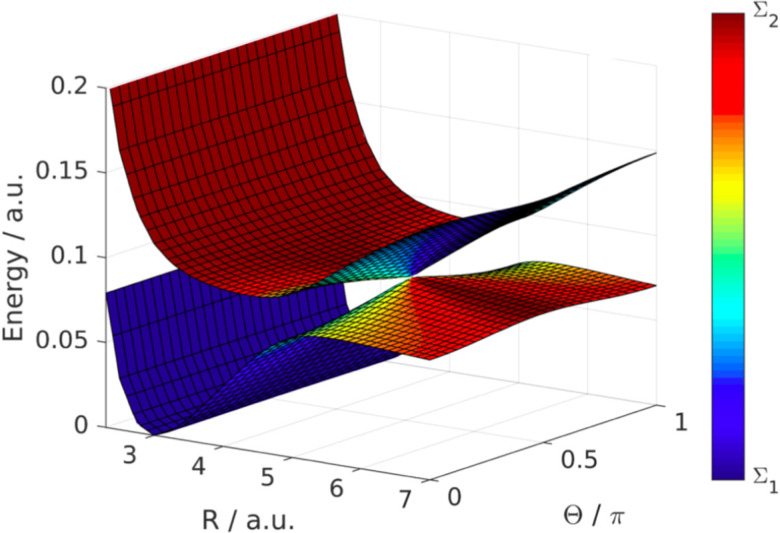
LICI between dressed states in the LiF molecule. The figure shows the PESs in dependence of the LiF bond length *R* and the angle between molecular axis and the electric field polarization *Θ*. The colour scale indicates the molecular character of the dressed states. The degeneracy appears at the point where the dressing field is resonant with the Σ_1_ and Σ_2_ state and the electric field polarization is orthogonal to the transition dipole moment (*R* = 5 bohr, *Θ* = 0.5π). Reproduced from ref. 129. Copyright 2019, IOP Creative Commons License CC BY 4.0.

The time-dependent Hamiltonian describing the coupled light-molecule system can generally be represented in the Floquet picture, which provides a simple way to explain the various chemical/physical effects of strong fields.^124–128^ The transformation of the full Hamiltonian into an equivalent time-independent version (Floquet Hamiltonian) involves the Fourier expansion of the time-dependent Schrödinger equation. This representation is particularly helpful to understand the occurrence of LICIs in diatomics. For instance, the Floquet Hamiltonian of the LiF molecule interacting with a laser field reads as follows:31
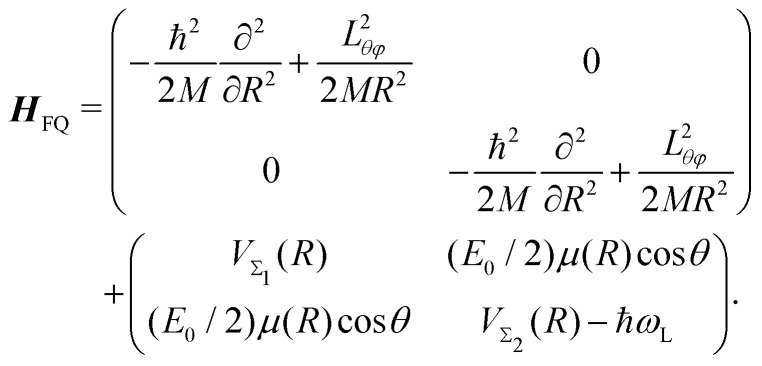
Here, the first matrix corresponds to the molecular ro-vibrational kinetic energy where *R* and (*θ*, *φ*) represent the vibrational and rotational coordinates, respectively. *M* is the reduced mass and *L*_*θφ*_ is the angular momentum operator. The second term represents the potential matrix, with *V*_*Σ*1_ and *V*_*Σ*2_ being the ground and excited potentials of the field-free LiF molecule, respectively. In this time-independent dressed-state picture, the light–matter interaction is described by shifting the excited state energy by *ħω*_L_, where *ω*_L_ denotes the laser frequency.^117^*E*_0_ represents the electric field amplitude, *I*_0_ (∝*E*_0_^2^) is the laser intensity, *μ*(*R*) is the transition dipole moment and the molecular rotational motion, *θ* is the angle between the polarization and the transition dipole. The potential matrix in eqn (31) is diagonalized to obtain the adiabatic potentials *V*_lower_(*R*,*θ*) and *V*_upper_(*R*,*θ*). The resulting adiabatic potentials of the LiF molecule are shown in Fig. 13. The adiabatic energy surfaces cross and form a LICI if the two conditions cos *θ* = 0 (*θ* = π/2) and *V*_*Σ*1_(*R*) = *V*_*Σ*2_(*R*) − *ħω*_L_ are satisfied simultaneously.

LICIs can alternatively also be formed by employing the quantized electromagnetic field in optical cavities.^129–135^ Currently, there exist numerous reports showing the influence of classical or quantum LICIs on chemical reactions such as photoisomerization, photodissociation, and electron transfer processes involving even polyatomic molecules.^136–148^ These studies indirectly provide the signatures of LICIs and demonstrate the great potential of the electromagnetic field as a control tool for photochemical processes. A direct mapping of such nonadiabatic phenomena, which is still limited,^136,149,150^ undoubtedly provides much deeper insights on strong light–matter chemistry. Impressive developments in the area of spectroscopic techniques observing the fingerprints of intrinsic CIs, as discussed in this feature, can be attributed in part to the remarkable growth of nonadiabatic field. In contrast to intrinsic CIs where the position and coupling strengths are inherent properties of the molecule, the location and coupling strength of LICIs can be controlled by using laser/cavity frequency and field intensity, respectively. This additional tuning may be advantageous for studying CI in greater detail.

## V. Summary and outlook

In conclusion, we have presented a theory overview over X-Ray/XUV spectroscopic and diffraction techniques, which may be used to gain deeper insight into the ultra-fast dynamics of CIs. A commonality between all schemes is that they are linear with respect to the detection scheme. In contrast to quadratic detection schemes, these schemes are directly sensitive to the phase of the molecular wave function. This allows one to explicitly detect electronic coherences created in the vicinity of a CI. The TXAS technique is experimentally well established and should, in principle, contain signatures of characteristic coherences. However, in practice the signatures are difficult to extract, and the signal mostly delivers the time evolution of populated electronic states involved. TRPES looks formally similar to TXAS. However, the target states, populated by the probe pulse, are not core excited states but rather ionic states in combination with free-electron states. Transitions from a neutral molecule to an ion provide more allowed transitions, than transitions between states in a neutral molecule, which may be restricted by symmetry-based selection rules.^151^ Moreover, theory and experiment have shown that TRPES signals exhibit coherent oscillations, when electronic coherences are present. This may allow a direct glimpse on the dynamics close to a CI with an experimentally well-established technique. In addition, XSES provides a unique feature: spectral features may draw a clear picture of CIs directly in the energy domain. In contrast to absorption-based signals, XSES does not depend on the populations of the valence states of interest. The TRUECARS technique is a linear Raman technique and thus is only sensitive to coherences, rather than populations, which sets it apart from the above-mentioned methods, by being background free with respect to the populations. The signal will in practice also contain contributions from vibrational coherences created by the UV pump-pulse which could be considered as unwanted background. However, it has been shown, that these contributions can be partly separated from the electronic coherences created by the CI.^86^ The TRUECARS technique has not yet been demonstrated experimentally, but is a promising candidate for a direct detection of nonadiabatic process near a CI. Time resolved XRD is not a spectroscopic technique and thus takes a special place among the techniques mentioned in this paper. However, it can provide further insight *via* the spatial domain rather than through the energy domain. We have shown that the diffraction pattern also contains information on the electronic coherences and molecular geometries at which these coherences have been created.

The techniques reviewed in this article provide access to the time evolving energy separation between the electronic states and, in some cases, also access to the phase of the electronic super position. The latter is important for the realisation of experiments that aim at observing the influence of the geometric phase in the vicinity of a CI. The geometric phase is well understood theoretically, but has never been directly observed in an experiment. A technique, such as TRUECARS, is thus an interesting candidate for future experiments that may shine light on the occurrence of a geometric phase. Three-state CIs have so far been only studied theoretically. While several studies clearly hint at their existence in commonly available molecules, it may be challenging to observe them in future experiments, as the branching of three states needs to be observed. The above-mentioned TRUECARS method as well as XSES may be good candidates, which are capable of disentangling a more complex branching scenario. Detailed studies of CIs in molecules can be difficult to carry out, depending on the electronic structure and location of the CI. A detailed picture of the shape of the CI requires a compact wave packet and thus favours smaller molecules and system where the CI is close to the Franck–Condon points. The shorter the time between the UV excitation and the interaction with a CI the better it can be resolved with the presented methods. Light induced CIs thus provide an interesting opportunity for studying basic features of CIs. The choice of photon energy, which creates the LICIs determines location and shape of the CI. Measurements of LICIs may thus also shine light on the mechanisms of governing photochemistry in the presence of optical nano-resonators, plasmonic structures, and dressing laser fields.

## Author contributions

T. S., D. J., and M. G. wrote the original draft. M. K. supervised the writing process and edited the manuscript.

## Conflicts of interest

There are no conflicts to declare.

## Supplementary Material

## Figures and Tables

**Fig. 14:**
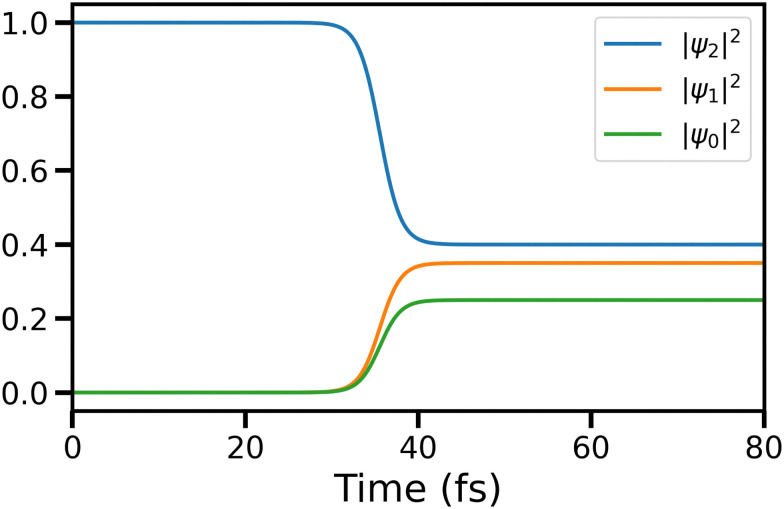
Population dynamics of states 0, 1, and 2 involved in the three-state CI are shown here.

## Appendices

### Appendix

#### Three-state CI

Time-dependent energies of electronic states involved in the three-state CI in atomic units (*ħ* = *e* = *m*_e_ = 4π*ε*_0_ = 1) for three nuclear dynamics in the system are given by,

32

where *t* represents time in atomic units. Time-dependent populations of electronic states before and after the ci are shown in Fig. 14.

The decoherence factor which is used to incorporate the decay of the coherence between the involved electronic states is given by,

33
